# Transcriptional expression of *PHR2* is positively controlled by the calcium signaling transcription factor Crz1 through its binding motif in the promoter

**DOI:** 10.1128/spectrum.01689-23

**Published:** 2023-12-06

**Authors:** Linghuo Jiang, Huihui Xu, Min Wei, Yiying Gu, Hongbo Yan, Lingxin Pan, Chunyu Wei

**Affiliations:** 1 Laboratory of Yeast Biology and Fermentation Technology, Institute of Biological Sciences and Technology, Guangxi Academy of Sciences, Nanning, Guangxi, China; 2 Department of Food Science, School of Agricultural Engineering and Food Science, Shandong University of Technology, Zibo, China; University of Wisconsin-Madison School of Medicine and Public Health, Madison, Wisconsin, USA

**Keywords:** *Candida albicans*, calcium, *PHR2*, Crz1

## Abstract

GH72 family of β-(1,3)-glucanosyltransferases is required for cell wall biogenesis, morphogenesis, and virulence of fungi. *Candida albicans PHR2* is an acidic pH-induced gene that encodes a GH72 enzyme involved in cross-linking of β−1,3- and β−1,6-glucans in the cell wall. In response to environmental pH changes, the expression of *PHR2* is negatively regulated by the transcription factor CaRim101 through the pH-responsive Rim101 pathway. In this study, we have generated the inactivation mutant for *PHR2* through the CRISPR/Cas9 approach. The *PHR2* mutant is sensitive to both membrane- and cell wall-disturbing agents, SDS, Calcofluor white and casponfungi, but resistant to two azole antifungals, ketoconazole and fluconazole. Although the *PHR2* mutant is not sensitive to calcium stress, it becomes sensitive to high levels of calcium stress in the presence of cyclosporin A, the specific inhibitor of calcineurin. In addition, we have demonstrated that the expression of *PHR2*, the downstream target of CaRim101, is positively controlled by the transcription factor CaCrz1 through its binding motif (5′ A_-315_TAGGCACAG 3′) in the *PHR2* promoter. Furthermore, the deletion of *PHR2* increases the cell precipitation rate in cell culture under acidic conditions.

**IMPORTANCE:**

The fungal cell wall consists of glucans, mannoproteins, and chitin and is essential for cell viability, morphogenesis, and pathogenesis. The enzymes of the GH72 family are responsible for ß-(1,3)-glucan elongation and branching, which is crucial for the formation of the glucan-chitin polymer at the bud neck of yeast cells. In the human fungal pathogen Candida albicans, there are five GH72 enzyme-encoding genes: *PHR1, PHR2, PHR3, PGA4*, and *PGA5*. It is known that expression of *PHR1* and *PHR2* is controlled by the pH-responsive Rim101 pathway through the transcription factor Rim101. In this study, we have demonstrated that the transcription expression of *PHR2* is also controlled by the transcription factor Crz1 through its binding motif in the promoter. Therefore, we have uncovered a dual-control mechanism by which *PHR2* expression is negatively regulated via CaRim101 through the pH-responsive pathway and positively modulated by CaCrz1 through the calcium/calcineurin signaling pathway.

## INTRODUCTION

As nutrients and signaling molecules, calcium ions regulate cell growth, differentiation, mating, and the synapses between neurons, as well as the initiation of the heartbeat and tasting in eukaryotes (1 – 4). Intracellular calcium homeostasis is maintained by calcium transporters and sequestrators in the plasma and organelle membranes, and regulation of intracellular calcium homeostasis and calcium/calcineurin signaling is highly conserved in eukaryotic cells (5 – 10). Transient increases in cytosolic Ca^2+^ activate the calcium/calcineurin signaling pathway through the calcium sensor calmodulin and the protein phosphatase calcineurin. Activated calcineurin dephosphorylates the transcription factor Crz1, which leads to its relocation from the cytosol to the nucleus, where it controls the expression of calcium-responsive genes in fungal cells (11, 12). Cells lacking *CRZ1* have less severe growth phenotypes and virulence than cells deleted from calcineurin, indicating that calcineurin has additional Crz1-independent cellular functions (13 – 16). Calcineurin regulates essential functions in the virulence of fungal pathogens, including adaptation to host temperature, hyphal growth, drug tolerance and resistance, cell wall integrity, and sexual development. This makes calcineurin an attractive target in fungal drug development (17). Another transcription factor, Crz2, is the homologue of Crz1, but not the target of calcineurin, in the most prevalent human fungal pathogen, *Candida albicans* (16, 18). However, previous studies have shown that Crz2 is involved in the regulation of pH response, early adaptation to murine gastro-intestine tract, cell adherence to silicone substrate, and biofilm formation in *C. albicans* (18 – 21).

*C. albicans* is normally present on mucosal surfaces of the oral, gastric, and genital systems in healthy individuals but causes systemic infections in immunocompromised patients (22 – 24). It has the ability to grow in the acidic stomach (pH 2.0) and vagina (pH 4.5), the neutral pH niches including the kidney, the liver, and the duodenum (pH 7.0), as well as the slightly alkaline blood (pH 7.3) (25, 26). Similar to *Saccharomyces cerevisiae*, *C. albicans* senses environmental pH changes through the Rim101 pathway, which is mediated by the transcription factor Rim101. Rim101 activates the expression of alkaline pH-induced genes such as *PHR1* and represses the expression of acidic pH-induced genes such as *PHR2* (27 – 29). The Rim101 pathway also plays a role in pH-induced filamentation and growth under alkaline conditions as well as in the response to lithium and hygromycin B in *C. albicans* (30 – 32). Previous studies have demonstrated that the Rim101 pathway is also involved in calcium stress independent of calcineurin in *S. cerevisiae* (33 – 36). Both the Rim101 pathway and the calcineurin/Crz1 pathway act in parallel to adapt to alkaline pH in *C. albicans* (18, 30).

The fungal cell wall consists of glucans, mannoproteins, and chitin, and is essential for cell viability, morphogenesis, and pathogenesis. Most of the major cell wall components of fungal pathogens are not present in mammals or plants, so the enzymes that assemble fungal cell wall components are excellent targets for antifungal drug development (37). The GH72 family of glucanosyltransferases is responsible for β-(1,3)-glucan elongation and branching, which is crucial for the formation of the glucan-chitin polymer at the bud neck of yeast cells (38 – 40). ScGas1 is a representative of the GH72 family, which also includes ScGas2, ScGas4, ScGas5, CaGas1, Phr1, and Phr2 (41, 42). Phr1 and Phr2 are involved in the cross-linking of β−1,3-glucan and β−1,6-glucan in the cell wall (43). Lack of *PHR1* leads to the loss of virulence of *C. albicans* cells in the mouse model of systemic infection but does not affect their virulence in the rat model of vaginal infection. In contrast, lack of *PHR2* does not affect lethality in mouse systemic infection but causes a loss of virulence of *C. albicans* cells in rat vaginal infection (44). A recent study shows that a lack of Phr1 triggers an adaptive response aimed at reinforcing the hyphal cell wall in *C. albicans* (38). Phr2 is required for the acidic growth of *C. albicans* cells (45).

In our recent work, we screened the GRACE (gene replacement and conditional expression) library of 2,358 conditional mutants and identified a total of 21 genes whose conditional repression leads to the sensitivity of *C. albicans* cells to calcium stress (46). Except of *CaCRZ1*, *CaMIT1* and *CaRCH1* (11, 47 – 49), this screen has identified additional 18 genes, including *CaRIM21*, involved in calcium sensitivity. *CaRIM21* encodes the plasma membrane pH sensor involved in the pH-responsive Rim101 pathway (50). In addition, expression of *PHR1* and *PHR2*, two downstream targets of CaRim101, is positively regulated by CaCrz1 (Fig. S1; GEO Accession number: GSE123122 (11)). In this study, we have demonstrated that the transcription expression of CaRim101-regulated *PHR2* is also controlled by the transcription factor Crz1 through its binding motif in the promoter.

## MATERIALS AND METHODS

### Strains and media

Strains, plasmids, and primers used in this study were listed in Table 1; Table S1, respectively. LB medium (10 g/L tryptone, 10 g/L NaCl, and 5 g/L yeast extract) was used for growing bacterial strains. YPD medium (20 g/L glucose, 20 g/L peptone, and 10 g/L yeast extract) and SD medium (0.67% yeast nitrogen base without amino acids, 2% glucose, and auxotrophic amino acids as needed) were used for growing *C. albicans* strains. For alkaline and acidic treatment, cells were grown in YPD +150 mM HEPES buffered at pH 8.0 and pH 4.0, respectively (11). Other media were not buffered unless indicated.

**TABLE 1:** Strains and plasmids used in this study

Name	Genotype or description	Source
Strain		
SN148	Mat*a/α arg4/arg4 leu2/leu2 his1/his1 ura3*::*imm434/ura3*::*imm434*	(51)
HHCA320	SN148 *phr2/phr2 ENO1/eno1:: natMX4*	This study
HHCA321	SN148 *phr2/phr2 ENO1/eno1:: natMX4*	This study
HHCA529	HHCA320 *RPS1/rps1::*CIp10	This study
HHCA531	HHCA320 *RPS1/rps1::*CIp10-PHR2	This study
HHCA752	SN148 *RPS1/rps1::*CIp10-PHR2-lacZ	This study
HHCA1194	SN148 *RPS1/rps1::*CIp10-PHR2(HΔ)-lacZ	This study
HHCA1	SN148 *RPS1/rps1::*CIp10	(11)
HHCA211	SN148 *crz1/crz1*	(11)
Plasmid		
pV1093	*Amp^R^ Nat^R^*	(52)
pV1093-sgPHR2	*Amp^R^ Nat^R^*	This study
CIp10	*Amp^R^ URA*	(53)
CIp10-sgPHR2	*Amp^R^ URA*	This study
CIp10-PHR2-lacZ	*Amp^R^ URA*	This study
CIp10-PHR2(HΔ)-lacZ	*Amp^R^ URA*	This study

Chemicals were purchased from Sigma (USA) and Sango Biotech (Shanghai, China). Stock solutions (1,000×) for ketoconazole, fluconazole, terbinafine, and tunicamycin were prepared in pure ethanol, and stock solutions for other chemicals/drugs used in this study were prepared in water.

### Construction of mutants for *PHR2* and *CRZ1*

CRISPR (Clustered Regularly Interspaced Short Palindromic Repeat/Cas9) approach was used for constructing inactivation mutants for *PHR2* in the *C. albicans* strain SN148 background as described previously [(11); Fig. S2]. Mutated sites in their CRISPR mutants were confirmed by DNA sequencing.

To construct the double-gene deletion mutant between *PHR2* and *CaCRZ1* in the CRISPR mutant for *PHR2*, we PCR amplified the *HIS1* and *ARG4* cassettes from pFA-HIS1 and pFA-ARG4 plasmids, respectively, to subsequently replace two alleles of *CaCRZ1* through the homologous recombination strategy (Fig. S3).

### Gene cloning

We cloned the full-length *PHR2* gene in the integration vector CIp10 as described (53). A DNA fragment containing the 705 bp promoter, the 1635 bp ORF, and the 370 bp terminator of *PHR2* was amplified with primers PHR2-clone-F and PHR2-clone-R and cloned between *Kpn*I and *Xho*I sites in the CIp10, which yielded CIp10-PHR2. Inserts in recombinant plasmids were confirmed by DNA sequencing. For the complementation experiment, this recombinant plasmid was *Stu*I-linearized and integrated into the *RPS1* loci of the CRISPR mutant strain for *PHR2*.

The *PHR2* promoter was amplified with a pair of primers PHR2_P(1 kb)_F and PHR2_P(1 kb)_R from the SN148 genomic DNA and cloned into the *Xho*I site of CIp10-lacZ-TACT1 to yield CIp10-PHR2-lacZ as described (11). To mutate the CaCrz1-binding motif predicted in our study, the 5′-AAA(−316) ATAGGCACAG(− 306)CCA-3′region of the *CaPHR2* promoter was mutated to be 5′-AAA(−316)ATTCTAGAAG(− 306)CCA-3′ (we designated this mutation as PHR2(HΔ)). This was generated by a fusion PCR strategy. We amplified the upstream (A) and downstream (B) fragments of the *PHR2* promoter with two pairs of primers: PHR2_P(1 kb)_F/PHR2(H)_R(lacZ) and PHR2(H)_F(lacZ)/PHR2_P(1 kb)_R, respectively. These two PCR fragments were then fused by PCR with the pair of primers PHR2_P(1 kb)_F/PHR2_P(1 kb)_R, and the fused product was cloned into the *Xho*I site of CIp10-lacZ-TACT1 to yield CIp10-PHR2(HΔ)-lacZ. Inserts in recombinant plasmids were confirmed by DNA sequencing.

### Protein purification and EMSA

The His6-tagged CaCrz1 was expressed and purified as described (11). Two double-stranded EMSA probes were made by annealing two pairs of oligonucleotides, EMSA PHR2-F/R PHR2(H) and PHR2(M), respectively, and labeled at the 3′ end with the DIG Gel Shift Kit (Roche) according to the manufacturer’s recommendations.

### Chromosomal immunoprecipitation (ChIP) assay

One allele of *CaCRZ1* in the wild-type SN148 strain was chromosomally and C-terminally HA-tagged, which generates the strain expressing CaCrz1-HA, and the wild-type SN148 strain with the untagged wild-type CaCrz1 was integrated with the CIp10 vector as the control strain (11). These two strains were exposed to 0.2 M CaCl2 for 1 h before their cells were collected and treated with formaldehyde. Whole-cell proteins were extracted, and immunoprecipitation was done with anti-HA monoclonal antibodies. Immunoprecipitated pellets were used as templates for PCR with the primer pairs ChIP_PHR2-F and ChIP_PHR2-R.

### Galactosidase activity assay

To measure the wild-type and the mutated *PHR2* promoter-driven β-galactosidase activities in the wild type SN148, we linearized the two plasmids containing the *lac*Z reporters for the wild-type and the mutated *PHR2* promoter with the *Stu*I enzyme and integrated them into the *RPS1* locus of the wild-type strain SN148 as described (11). We determined the β-galactosidase activity using the substrate o-Nitrophenyl-b-D-galactopyranoside (ONPG). The data are the mean ± SD of six independent experiments. Significant differences were analyzed by GraphPad Prism version 4.00. *P* values of < 0.05 were considered to be significant.

## RESULTS

### Deletion of *PHR2* leads to alterations in the sensitivity of *C. albicans* cells to disturbing agents of the cell membrane and the cell wall

**FIG 1:**
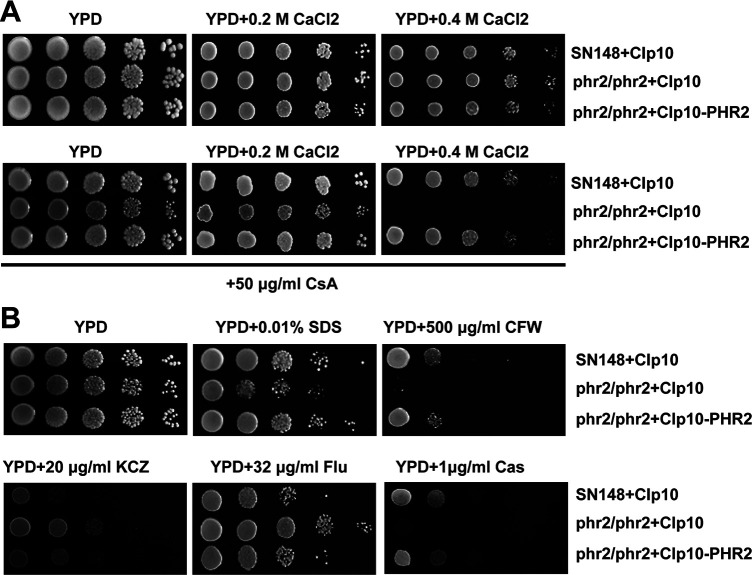
Roles of *PHR2* in the sensitivity to calcium (A) as well as to SDS, antifungal drugs, and cell wall-perturbing agents (B) *C. albicans* strains were grown overnight at 30°C in liquid SD-URA medium, and cultures were serially diluted 10 times and spotted onto YPD plates indicated. Plates were incubated at 30°C for 2–3 days before photos were taken. CFW, Calcofluor white; KCZ, ketoconazole; Teb, terbinafine; Flu, fluconazole; Cas, caspofungin.

*PHR2* is one of the downstream targets of the pH-responsive pathway transcription factor CaRim101 in *C. albicans* (28, 29). To examine if Phr2 is involved in the calcium sensitivity of *C. albicans*, we generated the inactivation homozygous mutant for *PHR2* through a CRSPR/Cas9 approach (Fig. S2). The homozygous mutant did not show a sensitive phenotype to both 0.2M CaCl_2_ and 0.4M CaCl_2_ in the absence of cyclosporin A (CsA), the specific inhibitor of calcineurin [Fig. 1A; (48)]. However, the homozygous mutant was sensitive to 0.4M CaCl_2_, albeit not to 0.2M CaCl_2_, in the presence of CsA, and this calcium sensitivity was reversed after the *PHR2* gene was introduced back to the homozygous mutant (Fig. 1A). This result indicates that Phr2 is required for *C. albicans* cells to adapt to a high level of calcium stress in the absence of functional calcineurin, but it is not essential for the response of *C. albicans* cells to calcium stress under normal conditions. In addition, the *phr2/phr2* mutant was sensitive to 0.01% SDS, 500 µg/mL Calcofluor white, and 1 µg/mL caspofungin, but tolerant to 20 µg/mL ketoconazole and 32 µg/mL fluconazole. These phenotypes can be reversed by the introduction of *PHR2* back to the homozygous mutant (Fig. 1B). No phenotype was observed for the *phr2/phr2* mutant in the presence of 0.1% ethanol (as a control), which was used to dissolve some of these tested drugs in the medium (data not shown).

### Deletion of *PHR2* reduces cell growth and increases cell precipitation rate in response to acidic stress

**FIG 2:**
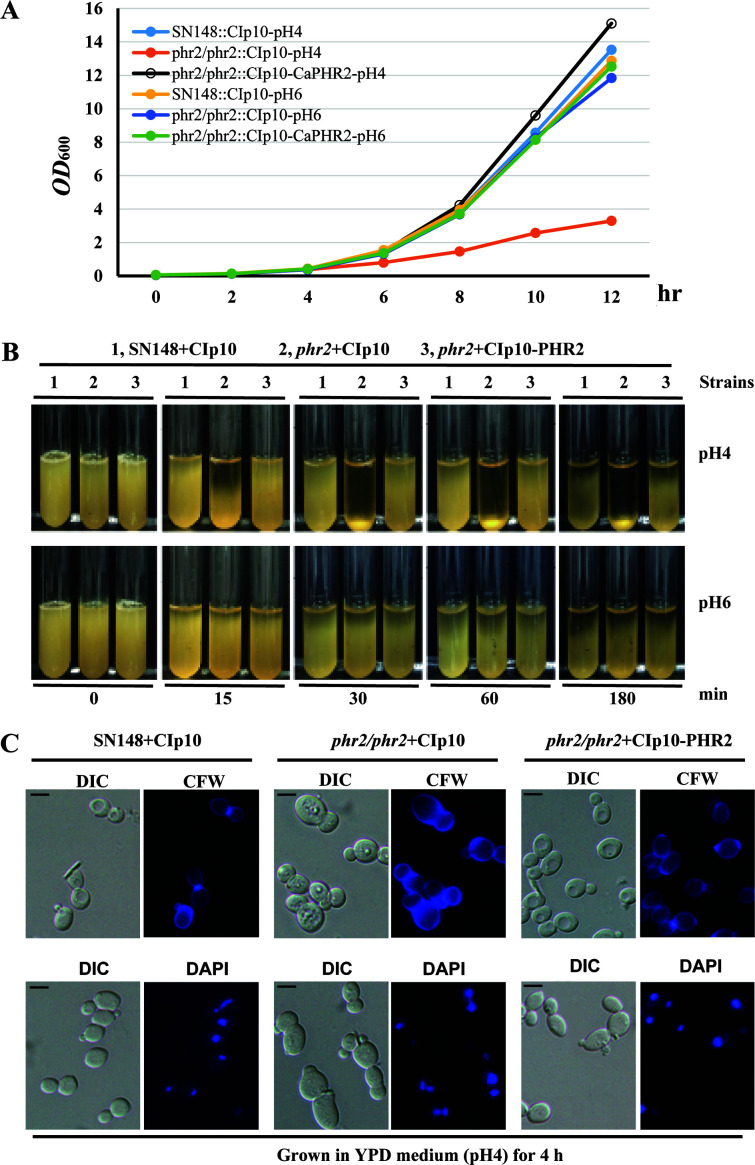
Functions of *PHR2* in cell growth (**A**), precipitation rate (**B**), constriction formation, and nuclear segregation (**C**). *C. albicans* strains were grown overnight at 30°C in liquid SD-URA medium, and cultures were inoculated and grown in YPD media with pH 4.0 or pH 6.0 for up to 12 h. Cell samples were taken every 2 h for the OD_600nm_ measurement (**A**). Log-phase growing cells of indicated strains in YPD media with pH 4.0 or pH 6.0 were allowed to precipitate in test tubes, and photos were taken at 0, 15, 30, 60, and 180 min (**B**). After log-phase growing cells of the indicated strains in YPD media with pH 4.0 were stained with Calcofluor white for chitin (top panel) or DAPI for nuclei (bottom panel), they were examined under conventional fluorescence microscopy (**C**). Scale bars, 5 µm.

*PHR2* is expressed at an acidic condition of pH 4.0, but its expression is repressed at pH 6.0 and more alkaline conditions (45). Consistently, we observed that *C. albicans* cells lacking a functional *PHR2* showed reduced growth at pH 4.0 but not at pH 6.0 as compared to the wild type and the complemented strain [Fig. 2A; (45)]. Interestingly, cells of the *phr2/phr2* mutant precipitated with a clear top phase formed in pH 4.0 culture 15 min after being mixed intensely, which is much faster than those of the wild type, and the complemented strains with a clear top phase formed 180 min afterward (Fig. 2B). However, when cultures were grown at pH 6.0, no difference was observed in cell precipitation rate among these three strains (Fig. 2B). This suggests deletion of *PHR2* increases cell precipitation rates under acidic conditions. Through microscopic observation, we showed that cells of the *phr2/phr2* mutant did not form aggregates or hyphae at pH 4.0 or pH 6.0, neither did the wild type or the complemented strain (Fig. S4). This suggests that the increased precipitation rate might be an issue of increased cell density rather than cell aggregation or hyphal formation for the *phr2/phr2* mutant at pH 4.0.

### Deletion of *PHR2* leads to a defect in the formation of constriction at the mother–daughter neck and nuclear segregation in response to acidic stress

To explore the nature of increased cell precipitation rate, we stained the cell wall with the chitin-binding agent, Calcofluor white. In the wild type and the complemented strains, chitin is normally distributed along the cell wall with a bright chitin ring formed at the narrow mother–daughter neck before cytokinesis (top panel in Fig. 2C). In contrast, the mother–daughter neck of the *phr2/phr2* mutant exhibited an abnormal enlargement of the neck constriction. Meanwhile, its cell size increased significantly (top panel in Fig. 2C). In addition, the *phr2/phr2* mutant cells showed mis-segregation of their nuclei, but cells of the wild type and the complemented strains did not (bottom panel in Fig. 2C). These phenotypes were not observed at neutral or alkaline pH conditions (data not shown). This suggests that Phr2 is required for the formation of constriction at the mother–daughter neck and proper nuclear segregation under acidic conditions.

### CaCrz1 binds *in vitro and in vivo* to the promoter of *PHR2*

**FIG 3:**
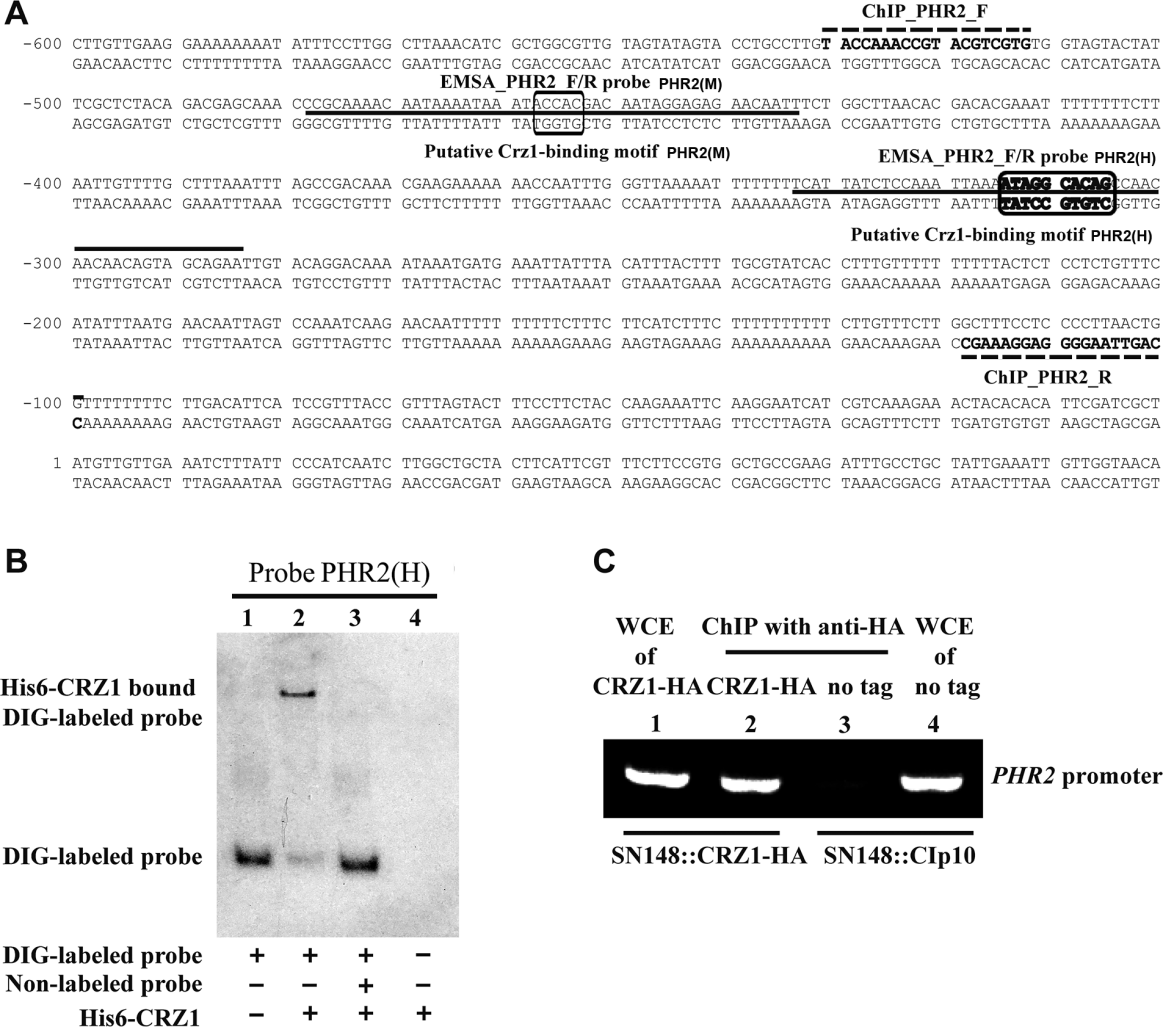
CaCrz1 binds *in vitro* and *in vivo* to the motif PHR2(H) in the promoter of *PHR2*. (**A**) Locations of two potential Crz1-binding motifs (boxed) in the *PHR2* promoter. The 5′-TGGTG-3′ region (corresponding to the 5′ A_-458_CCAC_-454_ 3′ region in the reverse complementary sequence) is the motif PHR2(M) predicted in the previous study (16), and the 5′ A_-315_TAGGCACAG_-306_ 3′ region is the motif PHR2(H) identified in another previous study (11). Locations of EMSA_PHR2_F/R probes are indicated with dark solid lines. Locations of the ChIP PCR primer pair [CHIP_PHR2_F and CHIP_PHR2_R] are indicated with broken lines. (**B**) DIG-labeled probe EMSA_PHR2_F/R [PHR2(H)] was added to samples in Lanes 1–3. Purified His6-Crz1 protein of 1 µg was added in Lanes 2, 3, and 4. Unlabeled probe PHR2(H) was added to samples in Lane 3. (**C**) Detection of Crz1 binding to the *PHR2* promoter *in vivo* by ChIP analysis. The wild-type SN148 strain expressing Crz1-HA and the control strain integrated with the CIp10 vector (no tag control) were grown to log-phase in the SD-URA medium, and cells were collected and treated with formaldehyde. Whole-cell extractions were obtained, and immunoprecipitation was done with anti-HA monoclonal antibodies. Immunoprecipitated pellets were used as templates for PCR with the primer pair ChIP_PHR2_F/R. PCR products were separated on a 1% agarose gel.

CaRim101 directly binds to the promoter of *PHR2* to repress its transcriptional expression in response to alkaline stress in *C. albicans* (28). Since we have observed that calcium-induced expression of *PHR2* is abolished in the absence of CaCrz1 (Fig. S1), we next examine if CaCrz1 directly binds to the promoter of *PHR2* to regulate its expression. Based on the consensus motifs [5′-GGAGGC(G/A)C(T/A)G-3′] and [5′-G(C/T)GGT-3′] in promoters of CaCrz1 target genes identified in two previous studies (11, 16), we analyzed the promoter sequence of *PHR2* and identified two potential CaCrz1-binding sites 5′ A_-315_TAGGCACAG 3′ (H) and 5′ G_-454_TGGT 3′ (M), respectively (Fig. 3A).

The EMSA assay showed that His6-CaCrz1 bound to the DIG-labeled PHR2_F/R PHR2(H) probe containing the potential CaCrz1-binding motif (H), but not to the DIG-labeled PHR2_F/R PHR2(M) probe containing the potential CaCrz1-binding motif (M), in the *PHR2* promoter. This (H) binding was eliminated by its specific competitor, the unlabeled EMSA_PHR2_F/R PHR2(H) probe (Fig. 3B; Fig. S5). These results demonstrate that CaCrz1 can indeed bind to the *PHR2* promoter *in vitro*.

To determine whether CaCrz1 binds to the *PHR2* promoter *in vivo*, we carried out ChIP experiments. After the 0.2M CaCl_2_ treatment for 1 hr, whole-cell extracts (WCEs) were prepared from log-phase growing cells expressing a functional chromosomally HA-tagged CaCrz1 (CaCrz1-HA) under the control of its own promoter (Lanes 1 and 2 in Fig. 3C) and cells with the untagged wild-type CaCrz1 (Lanes 3 and 4 in Fig. 3C). DNA samples isolated from their anti-HA chromatin immunoprecipitate were used in PCR assays to detect CaCrz1-HA target promoters (Lanes 2 and 3 in Fig. 3C). Their corresponding WCEs were used as controls in parallel PCR assays to ensure the equivalence of the IP starting material (Lanes 1 and 4 in Fig. 3C). We found that the DNA region covering the CaCrz1-binding motif (H) in the *PHR2* promoter was enriched in the anti-HA IPs of the CaCrz1-HA strain (Lane two in Fig. 3C), but not detected in the anti-HA IPs of the untagged CaCrz1 strain (Lane three in Fig. 3C). Together, these results demonstrate that CaCrz1 binds to the CaCrz1-binding motif (H) region of the *PHR2* promoter *in vivo*.

### Mutation of the putative Crz1-binding motif in the promoter of *PHR2* abolishes its calcium-induced expression

**FIG 4:**
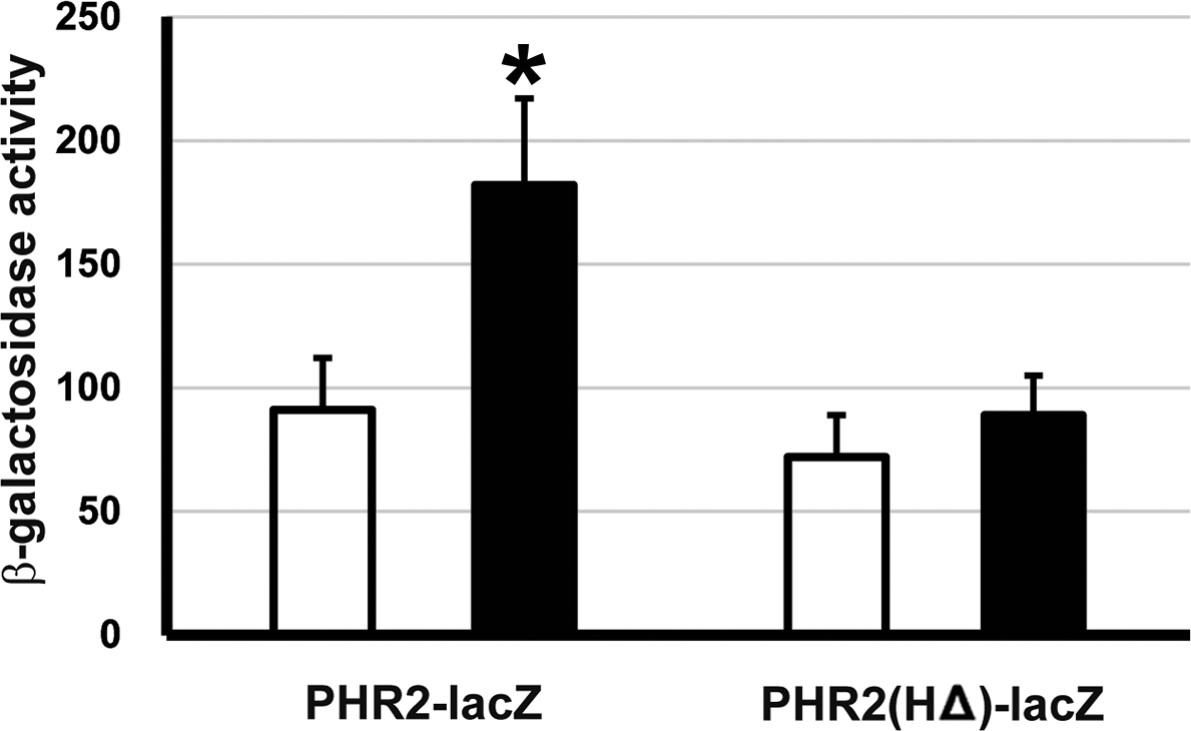
The Crz1-binding motif in the *PHR2* promoter plays a role in the regulation of *PHR2* expression. β-galactosidase activities of the wild-type promoter PHR2-*lac*Z and the mutated promoter PHR2(HΔ)-*lac*Z in the wild-type strain SN148 in the absence or presence of 0.2 M CaCl_2_. The asterisk (*) indicates the statistically significant difference (*P* < 0.05) in the β-galactosidase activity of the wild-type promoter in the wild-type SN148 cells between the absence and the presence of supplemented 0.2 M CaCl_2_.

To examine the effect of the Crz1-binding motif on the expression of *PHR2*, we introduced the two plasmids, CIp10-PHR2-lacZ and CIp10-PHR2(HΔ)-lacZ, containing the wild-type *PHR2* promoter, and the mutated *PHR2* promoter into the wild-type SN148 cells. In the absence of supplemented calcium, a basal level of β-galactosidase activity was detected for the wild-type promoter PHR2-lacZ in the wild-type cell, which was induced to increase by approximately two times in response to the treatment of 0.2M CaCl_2_ (left panel in Fig. 4). In contrast, a similar basal level of β-galactosidase activity was detected for the mutated promoter PHR2(HΔ)-lacZ in the wild-type cell, but it was not increased significantly in response to the treatment of 0.2M CaCl_2_ (right panel in Fig. 4). This result indicates that the calcium-induced expression of *PHR2* is dependent on the CaCrz1-binding site.

## DISCUSSION

**FIG 5:**
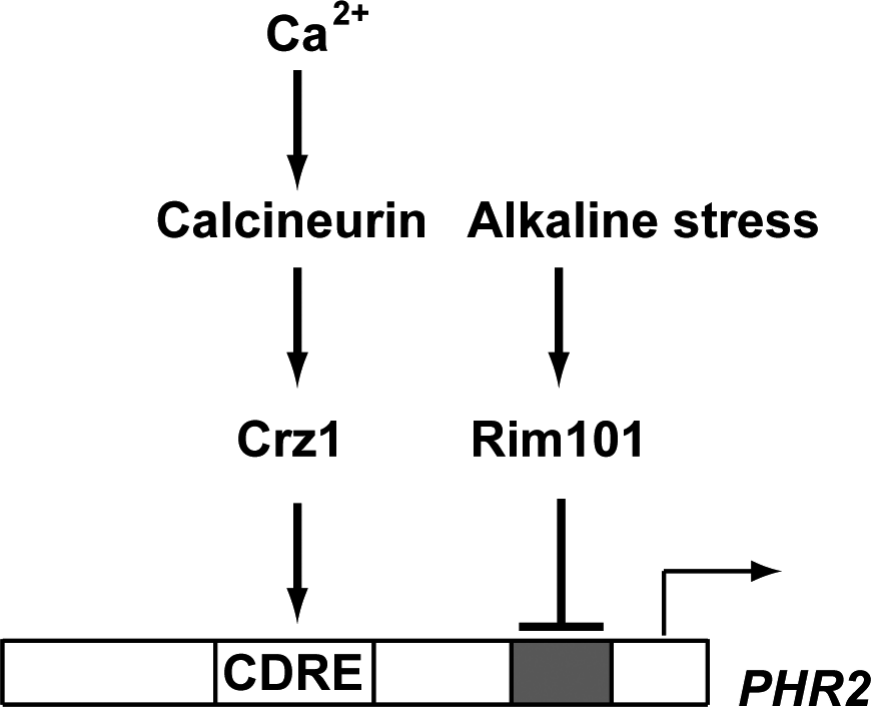
Schematic model. The positive and negative regulation of *PHR2* expression by the calcium/calcineurin signaling and the pH-responsive Rim101 pathways, respectively. Crz1-binding site (CDRE) and Rim101-binding site (shadowed) are indicated in the promoter of *PHR2*.

*C. albicans* is a commensal fungus of human mucosal surfaces that only causes disease in immunocompromised hosts. Its ability to adapt to diverse environmental pH within the host contributes to its success being both a commensal and a pathogen. This ability is controlled by the Rim101 signaling pathway. CaRim101 is a direct activator of *PHR1* transcription via the binding motif of 5′ CCAAGAAA 3′ and a direct repressor for *PHR2* expression mainly through the binding motif of 5′ GCCAAGAA 3′ (corresponding to its reverse complemented sequence 5′ T_-125_TCTTGGC 3′) (28, 29). The CaRim101 pathway acts in parallel to the calcium/calcineurin signaling pathway via the transcription factor CaCrz1 to adapt to alkaline pH (18). *PHR2* is one of the downstream targets of the CaRim101 pathway in *C. albicans* (24, 25). In this study, we show that *PHR2* is required for *C. albicans* cells to adapt to a high level of calcium stress in the absence of a functional calcineurin (Fig. 1). Transcription expression of *PHR2* is controlled by CaCrz1 through its binding motif 5′ A_-315_TAGGCACAG 3′ in the *PHR2* promoter (Fig. 3 and 4). Furthermore, we have shown in our recent study that Phr1 is required for the response of *C. albicans* cells to calcium stress, and calcium-induced expression of *PHR1* is also under the control of CaCrz1. More interestingly, alkaline- and calcium-induced Phr1 proteins are differentially glycosylated (54). Together with previous observations (18), these data support a dual-control model for transcription expression of both *PHR1* and *PHR2*, as exemplified here for *PHR2* (Fig. 5), by which *C. albicans* cells integrate both the Rim101 signaling and the calcium/calcineurin signaling to adapt to different pH environments. A similar dual-control model has also been observed previously for the transcription expression of *ScPMR1*, encoding the calcium pump on the ER/Golgi membrane, by the calcium/calcineurin signaling and the Rim101/Nrg1 pathway in *S. cerevisiae* (33, 55). It would be interesting to see whether the *PHR2* function in the calcium stress response is dependent on CaCrz1. We have tried a number of times to generate the double-gene mutant for *CaCRZ1* and *PHR2*. However, we were not successful in deleting two alleles of *CaCRZ1* in the CRISPR mutant of *PHR2* through the homologous recombination strategy (Fig. S3). This work remains to be carried out in our future study.

In addition, lack of *PHR2* leads to the sensitivity of *C. albicans* cells to disturbing agents of the cell membrane and the cell wall, but not lithium (Fig. 1; data not shown). In contrast, the deletion of *CaRIM13*, encoding the protease required for activation of CaRim101 via C-terminal cleavage, causes *C. albicans* cells sensitive to lithium (30). The Rim101 pathway also acts in parallel with Crz2 to adapt to lithium stress in a calcineurin-independent fashion (18, 30). In addition, lithium and antifungal drug sensitivity was observed for *C. albicans* lacking some components of the calcium/calcineurin signaling pathway in previous studies (56 – 58). Consistently, *C. albicans* lacking the negative regulator of calcium uptake CaRch1 are tolerant to lithium and antifungal drugs (48).

Expression of *PHR2* is progressively induced in more acidic conditions (45). Similar to previous observations in the CAF3-1 strain background, we have shown that deletion of *PHR2* in the SN148 strain background also leads to a defect in growth, the formation of constriction at the mother–daughter neck, and nuclear segregation in response to acidic stress [Fig. 2; (45)]. It is interesting to note that inhibition of septum formation by RO-09–3143, a specific inhibitor of Chs1 activity, in combination with *PHR1* deletion also leads to similar abnormal enlargement of the mother–daughter neck constriction and dramatic mis-segregation of the nuclei (38). Furthermore, *C. albicans* cells lacking *PHR2* exhibit an increased precipitation rate in the medium of pH 4.0, but do not show an increased precipitation rate in the medium of pH 6.0 (Fig. 2). This increased precipitation rate might be due to increased cell density rather than cell aggregation or hyphal formation for the cells lacking *PHR2* (Fig. S4). Our result is consistent with a previous study that deletion of *PHR2* does not affect the hyphal development in vaginal tissue with a pH of around 4.5 (44).

In conclusion, we have uncovered a mechanism by which *PHR2* expression is positively regulated via the transcription factor CaCrz1 through the calcium/calcineurin signaling pathway. CaCrz1 directly binds to the motif 5′ A_-315_TAGGCACAG 3′ in the promoter of the *PHR2* gene. This provides the molecular explanation by which *C. albicans* cells integrate both the Rim101 signaling and the calcium/calcineurin signaling pathways to adapt to different pH environments.

## SUPPLEMENTAL MATERIAL

Figure S1 spectrum.01689-23-s0001.pdf Transcript levels of the *PHR2* gene in the wild type SN148 and its isogenic mutant *crz1/crz1* cells.

Figure S2 spectrum.01689-23-s0002.pdf
*Candida albicans* CRISPR expression system for construction of the *phr2/phr2* mutant.

Figure S3 spectrum.01689-23-s0003.pdf Knockout strategy of two alleles of *CRZ1* in the CRISPR mutant for *PHR2* and PCR confirmation of genotypes.

Figure S4 spectrum.01689-23-s0004.pdf Cell morphology of the wild type SN148 + CIp10, the *phr2/phr2* + CIp10, and the *phr2/phr2* + CIp10-PHR2 strains under pH4 and pH6 conditions.

Figure S5 spectrum.01689-23-s0005.pdf CaCrz1 does not bind *in vitro* to the motif PHR2(M) in the promoter of *PHR2*.

Table S1 spectrum.01689-23-s0006.docx Primers used in this study.

ASM does not own the copyrights to Supplemental Material that may be linked to, or accessed through, an article. The authors have granted ASM a non-exclusive, world-wide license to publish the Supplemental Material files. Please contact the corresponding author directly for reuse.
