# Supplementary material for: Transcriptional expression of PHR2 is positively controlled by the calcium signaling transcription factor Crz1 through its binding motif in the promoter
Source: Microbiol Spectr. 2023 Dec 6;12(1):e01689-23. doi: 10.1128/spectrum.01689-23 (PMC10783099; doi:10.1128/spectrum.01689-23)
Supplement: Figure S1 — Transcript levels of the PHR2 gene in the wild type SN148 and its isogenic mutant crz1/crz1 cells. [file spectrum.01689-23-s0001.pdf]

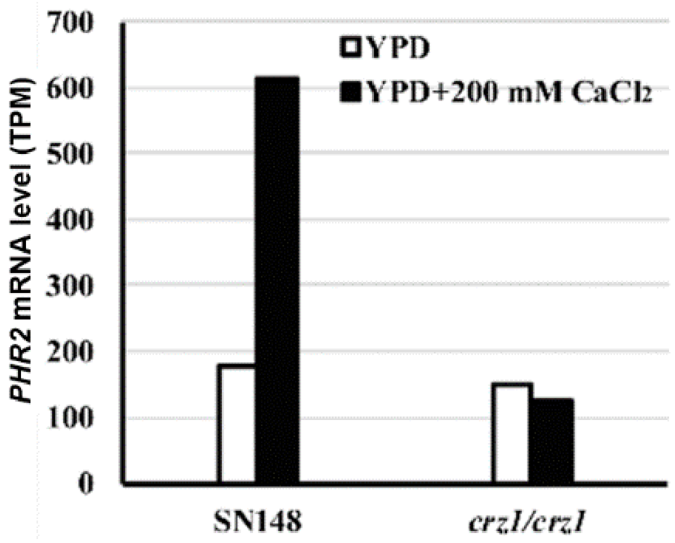

Figure S1. Transcript levels of the *PHR2* gene in the wild type SN148 and its isogenic mutant *crz1/crz1* cells growing in log phase in the presence or absence of 0.2M $CaCl_2$ for 2 hours. Data is from our previous study ([11]; GEO Accession number: GSE123122).
