# Supplementary material for: Transcriptional expression of PHR2 is positively controlled by the calcium signaling transcription factor Crz1 through its binding motif in the promoter
Source: Microbiol Spectr. 2023 Dec 6;12(1):e01689-23. doi: 10.1128/spectrum.01689-23 (PMC10783099; doi:10.1128/spectrum.01689-23)
Supplement: Figure S2 — Candida albicans CRISPR expression system for construction of the phr2/phr2 mutant. [file spectrum.01689-23-s0002.pdf]

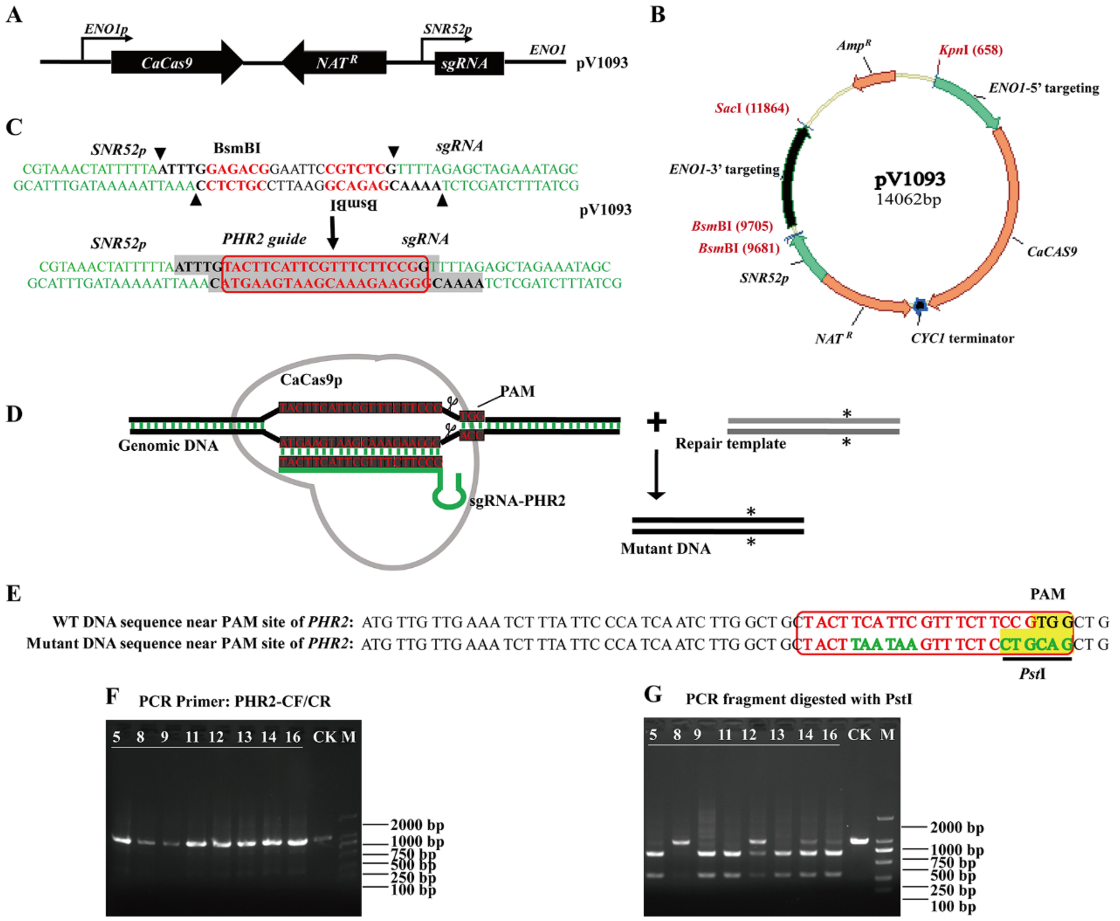

Figure S2. ***Candida albicans*** **CRISPR expression system for construction of** ***the phr2/phr2*** **mutant.** (**A**) Solo system consists of one plasmid, pV1093, which targets *ENO1*. (**B**) Profile of plasmid pV1093. The Cas9 gene is fused to sequences encoding the 3x SV40 nuclear localization signal and 3x FLAG tag for in-frame fusion to the 3' end of the gene. The Cas9 in this construct is expressed from the constitutive *ENO1* promoter at the plasmid integration site. The RNA polymerase III (Pol III) promoter *SNR52p* was used to express sgRNAs. (**C**) Guide expression system permits rapid cloning by digestion with *BsmB*I followed by ligation of annealed oligos (shaded sequences) with desired guide sequence (*PHR2* guide sequence in red box). (**D**) Schematic of Cas9 mutagenesis method. This system can create homozygous mutations in the gene (*, PAM site) and simultaneously mutate sequences to prevent repeated cleavage subsequent to integration. (**E**) DNA sequences of the *PHR2* locus in the wild-type and its isogenic mutant *phr1/phr1*. Two consecutive stop codons are in frame with the *PHR2* ORF. *Pst*I restriction enzyme site is introduced at PAM region. (**F**) PCR amplification of the 1-kb DNA region covering the mutated site within the *PHR2* locus from mutant transformants and the wild type (CK). (**G**) Digestion of corresponding PCR products in (F) by the restriction enzyme *Pst*I, whose site was introduced during the mutagenesis. Correct mutant transformants No. 5, No. 9, No. 11 and No. 13 were selected for DNA sequencing confirmation.
