# Supplementary material for: Transcriptional expression of PHR2 is positively controlled by the calcium signaling transcription factor Crz1 through its binding motif in the promoter
Source: Microbiol Spectr. 2023 Dec 6;12(1):e01689-23. doi: 10.1128/spectrum.01689-23 (PMC10783099; doi:10.1128/spectrum.01689-23)
Supplement: Figure S3 — Knockout strategy of two alleles of CRZ1 in the CRISPR mutant for PHR2 and PCR confirmation of genotypes. [file spectrum.01689-23-s0003.pdf]

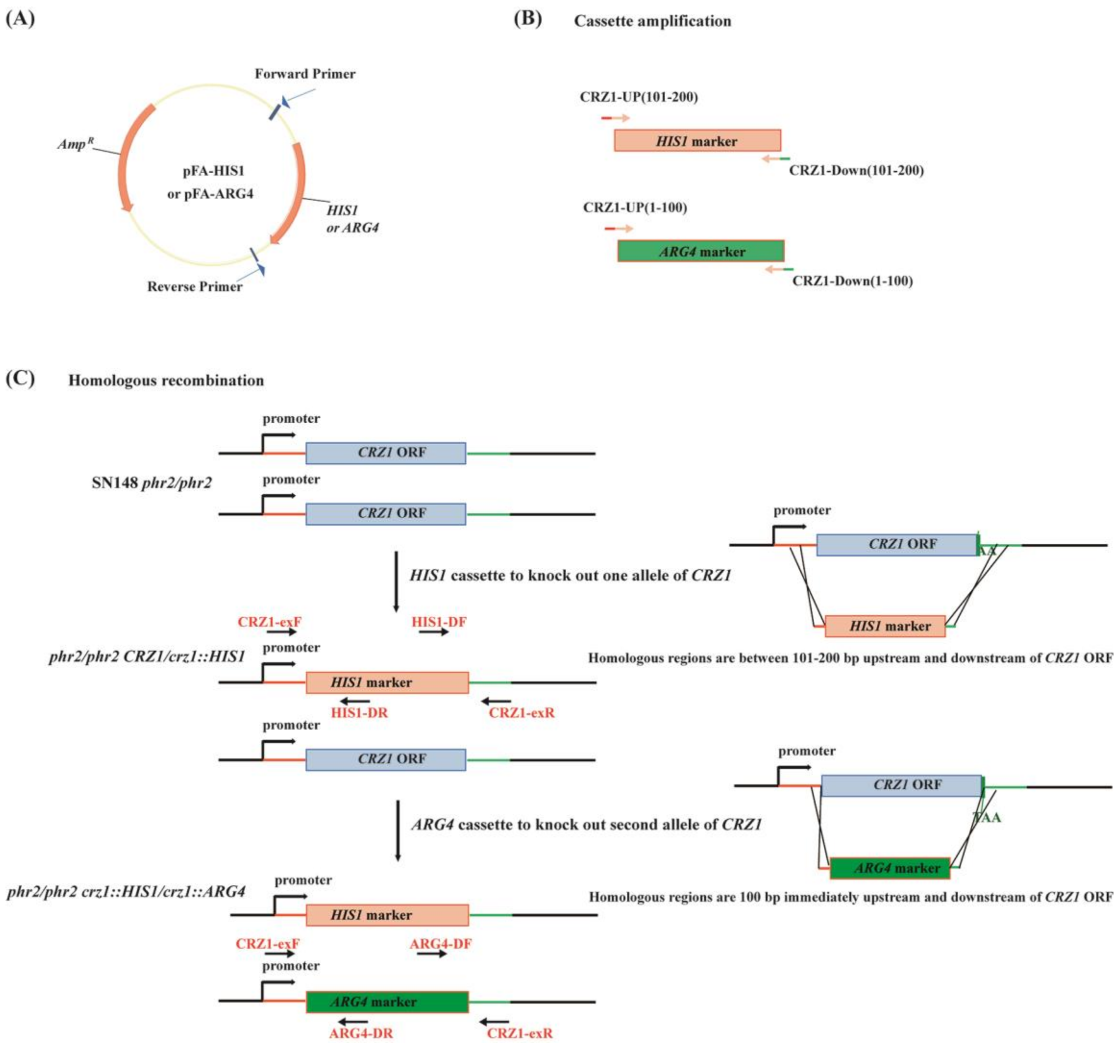

Figure S3. Knockout strategy of two alleles of *CRZ1* in the CRISPR mutant for *PHR2* and PCR confirmation of genotypes. A, Restriction maps of pFA-ARG4 and pFA-HIS1 plasmids. **B,** PCR amplification of *ARG4* and *HIS1* cassettes. **C,** knockout steps and PCR primers for confirmation of genotypes of homozygous mutants for *CRZ1*.
