# Supplementary material for: Transcriptional expression of PHR2 is positively controlled by the calcium signaling transcription factor Crz1 through its binding motif in the promoter
Source: Microbiol Spectr. 2023 Dec 6;12(1):e01689-23. doi: 10.1128/spectrum.01689-23 (PMC10783099; doi:10.1128/spectrum.01689-23)
Supplement: Figure S4 — Cell morphology of the wild type SN148 + CIp10, the phr2/phr2 + CIp10, and the phr2/phr2 + CIp10-PHR2 strains under pH4 and pH6 conditions. [file spectrum.01689-23-s0004.pdf]

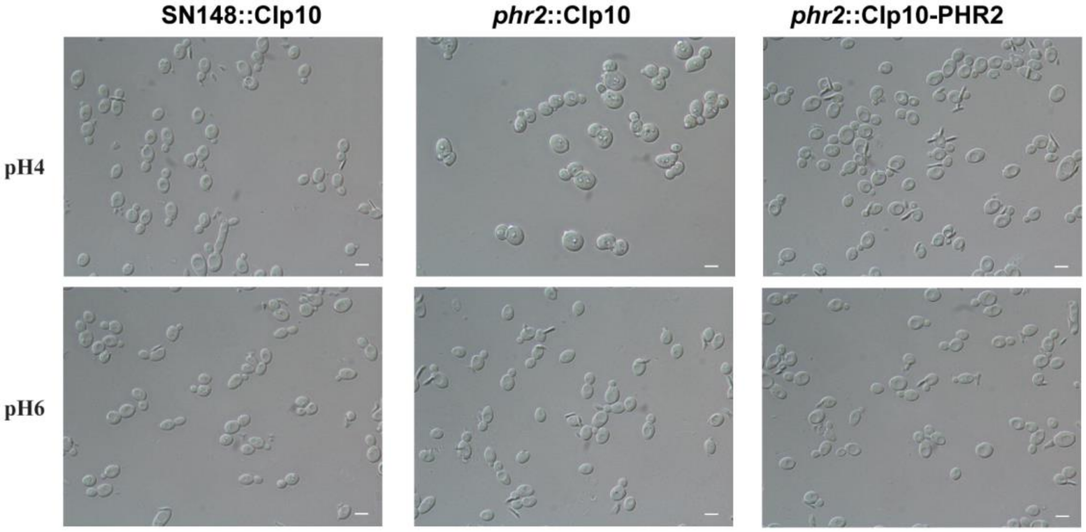

Figure S4. Cell morphology of the wild type SN148 + CIp10, the *phr2/phr2* + CIp10 and the *phr2/phr2* + CIp10-PHR2 strains under pH4 and pH6 conditions. *C. albicans* strains were grown overnight at 30°C in liquid SD-URA medium, and cultures were inoculated and grown in YPD media with pH4.0 or pH6.0 for 4 hours. Cell samples were examined under microscopy. Scale bars, 5 μm.
