# Supplementary material for: Transcriptional expression of PHR2 is positively controlled by the calcium signaling transcription factor Crz1 through its binding motif in the promoter
Source: Microbiol Spectr. 2023 Dec 6;12(1):e01689-23. doi: 10.1128/spectrum.01689-23 (PMC10783099; doi:10.1128/spectrum.01689-23)
Supplement: Figure S5 — CaCrz1 does not bind in vitro to the motif PHR2(M) in the promoter of PHR2. [file spectrum.01689-23-s0005.pdf]

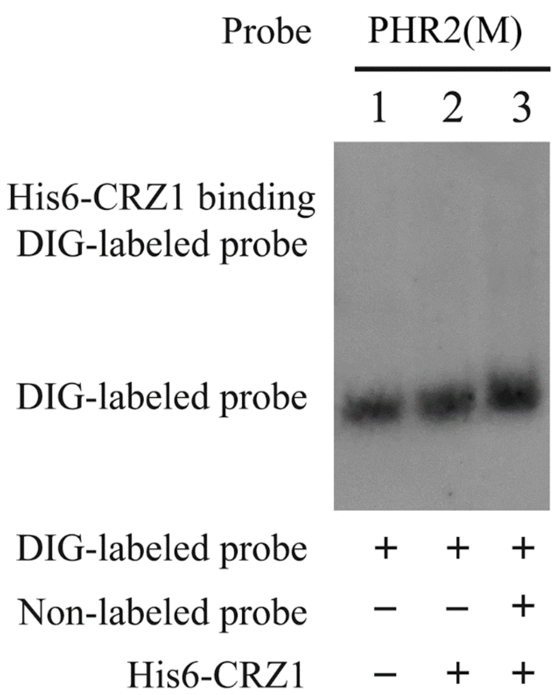

Figure S5. CaCrz1 does not bind *in vitro* to the motif PHR2(M) in the promoter of *PHR2*. EMSA analysis of His6-Crz1 binding to the potential Crz1-binding motif 5’ $G_{(454)}$TGGT 3’ (M) in the *PHR2* promoter. DIG-labelled probe PHR2(M) was added into samples in Lanes 1-3. Purified His6-Crz1 protein of 1µg was added into Lanes 2 and 3. Unlabelled probe PHR2(M) was added into samples in Lane 3.
