# Supplementary material for: Transcriptional expression of PHR2 is positively controlled by the calcium signaling transcription factor Crz1 through its binding motif in the promoter
Source: Microbiol Spectr. 2023 Dec 6;12(1):e01689-23. doi: 10.1128/spectrum.01689-23 (PMC10783099; doi:10.1128/spectrum.01689-23)
Supplement: Table S1 — Primers used in this study. [file spectrum.01689-23-s0006.docx]

**Table S1. Primer s used in this study**

| **Primer name** | **Sequence ( 5’-3’ )** |
| --- | --- |
| PHR2-sgF | cgtaaactatttttaatttgTACTTCATTCGTTTCTTCCG |
| PHR2-sgR | gctatttctagctctaaaacCGGAAGAAACGAATGAAGTAc |
| PHR2-RF | ctatgttgttgaaatctttattcccatcaatcttggctgcTACT**TAATAA**GTTTCTC**CTGCAG**CTG |
| PHR2-RR | aatttgttaccaacaatttcaatagcaggcaaatcttcggCAG**CTGCAG**GAGAAAC**TTATTA**AGTA |
| PHR2-CF | GGGAAGAAGTGGTAACATGTC |
| PHR2-CR | GATGGATTCATCTGGTTGGG |
| PHR2-clone-F | agggaacaaaagctgggtaccGGTTGGGGTGTTGTAGTTGCTG |
| PHR2-clone-R | atcgataccgtcgacctcgagCTTCCTCCATTTCATTGAGCTGG |
| LR159F | CCAAGAAGCATCTAATCAACTCCC |
| CRZ1-UP(1-100) | AATAGAACAA TTTAATCTGG TCATTTCATT TCCCCTAACA GCATCTTTCC AAGTTCAAAT ATTTTCCCCT TTTTATATCT AAATTTCATA AATCCCAATC gaagcttcgtacgctgcaggtc |
| CRZ1-Down(1-100) | AAATCATTCG TAAAACCAAA AGTAAAATAG AATAAAAAAA CAACCAACCA ACCAACCAAC AGGAATAACT ATCGTGAATG ACAACAACCT CAAAAAAAAA tctgatatcatcgatgaattcgag |
| CRZ1-UP(101-200) | CAAGCTAATC AAGCTTACCA AGCTATTTTT ATTGTTGATA TTGTTTTGTT TTCCCTTGAC AACAACGAAA AATTTCTTAT AATTCGACTA AACATAAAAA gaagcttcgtacgctgcaggtc |
| CRZ1-Down(101-200) | TTCAATTATG GAAAGTCCAT TTATATAATA CACAAGAACA ATAATAATAA ATAAATACAA AAGATATGAA AAAAAAAAAA GTATAAATGT CAAAAATAAA tctgatatcatcgatgaattcgag |
| CRZ1-exF | TTATTGAAGATACTAGC TTTCAAC |
| CRZ1-exR | TGTGTGTGTGCGTGTGTGTG |
| HIS1-DF | TTTAGTCAATCATTTACCAGACCG |
| HIS1-DR | ggttgcaccagctttcttc |
| ARG4-DF | ggatatgttggctactgatttagc |
| ARG4-DR | gtatgaatatcctcatcaccagcc |
| PHR2-P(1kb)-F | atcgataccgtcgacctcgagCGTCGTGTGGATCGAGTGG |
| PHR2-P(1kb)-R | ttcagtcatagccatctcgagAGCGATCGAATGTGTGTAGTTTCT |
| PHR2(H)-R(lacZ) | TGCTACTGTTGTTGTTGGCTTCTAGAATTTTAATTTGGAGATAATGAAAAAAAATTTTTAACCC |
| PHR2(H)-F(lacZ) | ATTCTAGAAGCCAACAACAACAGTAGCAGAATTG |
